# Top3α Is Required during the Convergent Migration Step of Double Holliday Junction Dissolution

**DOI:** 10.1371/journal.pone.0083582

**Published:** 2014-01-02

**Authors:** Stefanie Hartman Chen, Jody L. Plank, Smaranda Willcox, Jack D. Griffith, Tao-shih Hsieh

**Affiliations:** 1 Department of Biochemistry, Duke University Medical Center, Durham, North Carolina, United States of America; 2 Department of Microbiology, University of California Davis, Davis, California, United States of America; 3 Lineberger Cancer Center, University of North Carolina, Chapel Hill, North Carolina, United States of America; 4 Institute of Cellular and Organismic Biology, Academia Sinica, Taipei, Taiwan; Institut Pasteur, France

## Abstract

Although Blm and Top3α are known to form a minimal dissolvasome that can uniquely undo a double Holliday junction structure, the details of the mechanism remain unknown. It was originally suggested that Blm acts first to create a hemicatenane structure from branch migration of the junctions, followed by Top3α performing strand passage to decatenate the interlocking single strands. Recent evidence suggests that Top3α may also be important for assisting in the migration of the junctions. Using a mismatch-dHJ substrate (MM-DHJS) and eukaryotic Top1 (in place of Top3α), we show that the presence of a topoisomerase is required for Blm to substantially migrate a topologically constrained Holliday junction. When investigated by electron microscopy, these migrated structures did not resemble a hemicatenane. However, when Blm is together with Top3α, the dissolution reaction is processive with no pausing at a partially migrated structure. Potential mechanisms are discussed.

## Introduction

Double-strand DNA breaks, which occur frequently from irradiation, UV light, or stalled replication forks, are often repaired through homologous recombination, a process that is mostly error-free [1]. Following strand invasion of the broken end and synthesis off of the invaded strand, the ends can re-join to create an intertwined double Holliday junction (dHJ) structure [2], [3]. Although structure-specific resolvases can resolve these structures, the Blm-Top3α pathway plays a major role by dissolving these structures into non-crossover products, thereby preventing gene cluster rearrangements and loss-of-heterozygosity [4], [5]. Blm and Top3α have been shown to convergently migrate dHJ substrates *in vitro* [6], [7], but many details of the mechanism are not yet known.

Several groups have proposed that dHJ dissolution is a sequential mechanism [8], [9], with each enzyme performing one step. In the first step, Blm alone is proposed to convergently migrate the junctions into a hemicatenane structure, where only a single crossover of single strands remains between the two duplexes. In the second step, Top3α is recruited and decatenates the two strands, thereby separating the duplexes. These proposed steps are based on the individual mechanisms of the enzymes, which are able to branch migrate single HJ's [10] and decatenate single-strand DNA [11], respectively. However, the topological constraints imposed by the duplex connection between the two junctions likely present unique problems not encountered by these individual reactions.

Using the sequential mechanism as a hypothesis, the mechanism of dHJ dissolution was investigated. We found that Top3α does show preferential binding to the junction on a dHJ substrate, similar to Blm. In addition, migration of the junction requires the presence of a topoisomerase, although Top1 could substitute and allow limited migration. The migration product of Blm and Top1 was directly observed by electron microscopy, and the junctions remained 10's of base pairs (bp) apart, rather than converging at a hemicatenane. However, the dissolution reaction proceeds through both migration and decatenation with no apparent pause when both Top3α and Blm are present.

## Materials and Methods

### Protein purification

Recombinant *Drosophila* Blm and Top3α were purified as in [12]. Recombinant *Drosophila* Top1 was purified as in [13].

### Double Holliday junction substrate

The double Holliday junction DNA substrate and the mismatch double Holliday junction DNA substrate were created as in [14]. Dissolution reactions were performed as in [15].

### Electron microscopy

DNA samples (∼100 ng) were incubated with Blm (30 nM) and/or Top3α (50 nM) as appropriate. The reactions were performed in dissolution buffer (40 mM Tris-Cl pH 7.5, 0.1 mM EDTA, 4 mM MgCl2, 50 mM NaAc, 1 mM ATP). For reactions looking at the binding of Blm alone on DNA, ATP was excluded. The protein-DNA complexes were fixed with 0.6% glutaraldehyde (v/v) for 10 min at room temperature followed by gel filtration using a 1 mL Bio-Gel A-5m (BioRad) column to remove free protein and fixatives. No cross-linking step was used to treat protein-free samples following the reactions. The reactions were first stopped and the reaction products were digested with proteinase K at 55°C for at least two hours prior to column purification.

The samples were prepared for electron microscopy as described previously [16]. Briefly, the samples were adsorbed to thin carbon foils supported by 400-mesh copper grids in the presence of spermidine, then washed with a water/ethanol series, air dried, and rotary shadowcast with tungsten. The grids were visualized in a FEI T12 TEM instrument at 40 kV. Images for publication were captured using a Gatan CCD camera and measurements were taken using Digital Micrograph software (Gatan, Inc.)

## Results

### Top3α prefers binding to open Holliday junctions

Previous studies have shown that Blm is needed to direct Top3α to nuclear foci in unperturbed cells [17], [18], [19]. However, in *Xenopus*, Xtop3α was able to localize to chromatin when replication was inhibited even in the absence of Xblm [20], indicating a preference for DNA damage structures. Initial studies indicated that Top3α displays no preference for HJ over duplex oligonucleotide-based structures [15].

**Figure 1:**
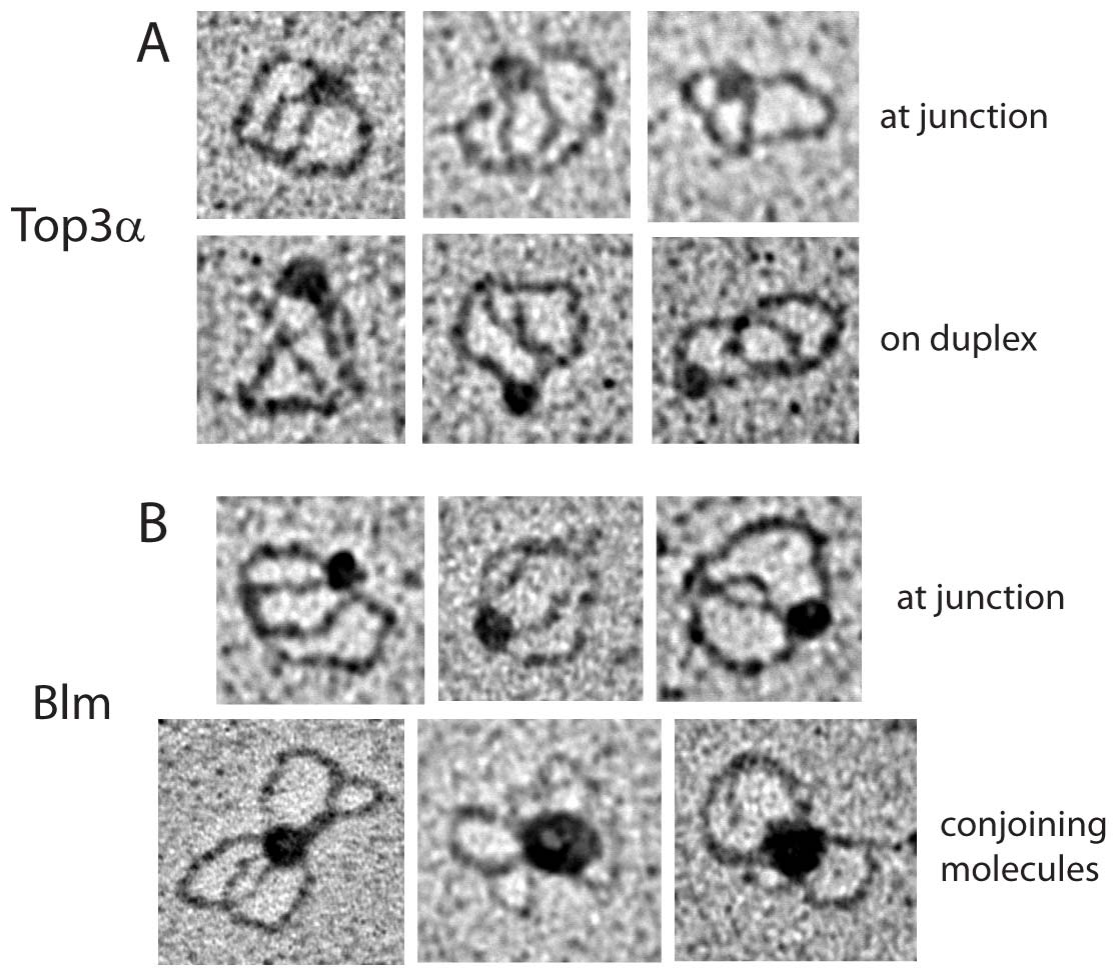
Electron microscopy of proteins on the dHJ substrate. A. Top3α bound to junction and non-junction sites. B. Blm bound at junctions and forming larger complexes with multiple DNA monomers. Enzymes were incubated with the DNA in dissolution buffer, in the absence of ATP to prevent helicase activity.

We tested whether Top3α bound preferentially on the dHJ substrate. Due to the lack of free ends for radiolabeling and a desire to distinguish between binding on different areas of the substrate, we employed electron microscopy to directly visualize the binding. Top3α was incubated with the substrate, fixed with 0.6% glutaraldehyde, passed over a gel filtration column, and mounted onto a carbon grid. Surprisingly, visualization of 83 molecules revealed that Top3α had a distinct preference for the junction site, localizing to the junction in 71% of the protein-bound DNA molecules (Fig. 1, Table 1). Many studies have indicated that HJ's with free ends are in the stacked, antiparallel conformation [21], [22], [23], while the HJ's in our dHJ substrate are necessarily parallel and in a more open conformation [14]. This difference in conformation likely accounts for the difference in Top3α binding preference (see Discussion).

**Table 1:** Occupancy of enzymes on the dHJ substrate, as visualized by electron microscopy.

	On duplex arm	At junction	At both junctions
Top3α	24 (28.9%)	59 (71.1%)	-
Blm	13 (17.1%)	61 (80.3%)	2 (2.6%)

Both Top3α and Blm prefer to bind at the junction site of the dHJ substrate.

As expected from previous work [10], Blm prefers binding at the junction site of the dHJ substrate (Fig. 1, Table 1). Over 80% of observed molecules had Blm localized to the junction. The size of the bound Blm complex was estimated by measuring the diameter and comparing it to the measured Stokes radius of purified protein [24]. Bound Blm at the junctions was found to be in the monomer/dimer range. This suggests that, unlike RuvAB [25], Blm does not operate as a hexamer to migrate junctions. In addition, despite an excess of Blm protein to DNA molecules, Blm almost always bound to only one of the two junctions on the substrate. This may be due to limiting glutaraldehyde during the crosslinking reaction, or may indicate that Blm prefers to bind only one junction and migrate it towards the other.

### Migration of constrained Holliday junctions requires a topoisomerase

Dissolution of the dHJ requires that the two junctions are convergently migrated and the two conjoined duplexes are then separated. It has been suggested that Blm alone can migrate the junctions together to create a hemicatenane structure, which is then resolved by Top3α. Although Blm is very efficient at migrating HJ's with free ends [10], the topological constraint of a double HJ may not allow such free migration. To test this, we utilized the mismatch dHJ substrate (MM-DHJS) [7], which contains disrupted restriction enzyme digestion sites that are restored when successful migration has occurred. The restriction digest can thus allow us to determine if the migration has moved beyond the mismatch sites. Migration can occur in the absence of full dissolution, as would occur in the creation of a hemicatenane, while dissolution necessarily requires migration.

**Figure 2:**
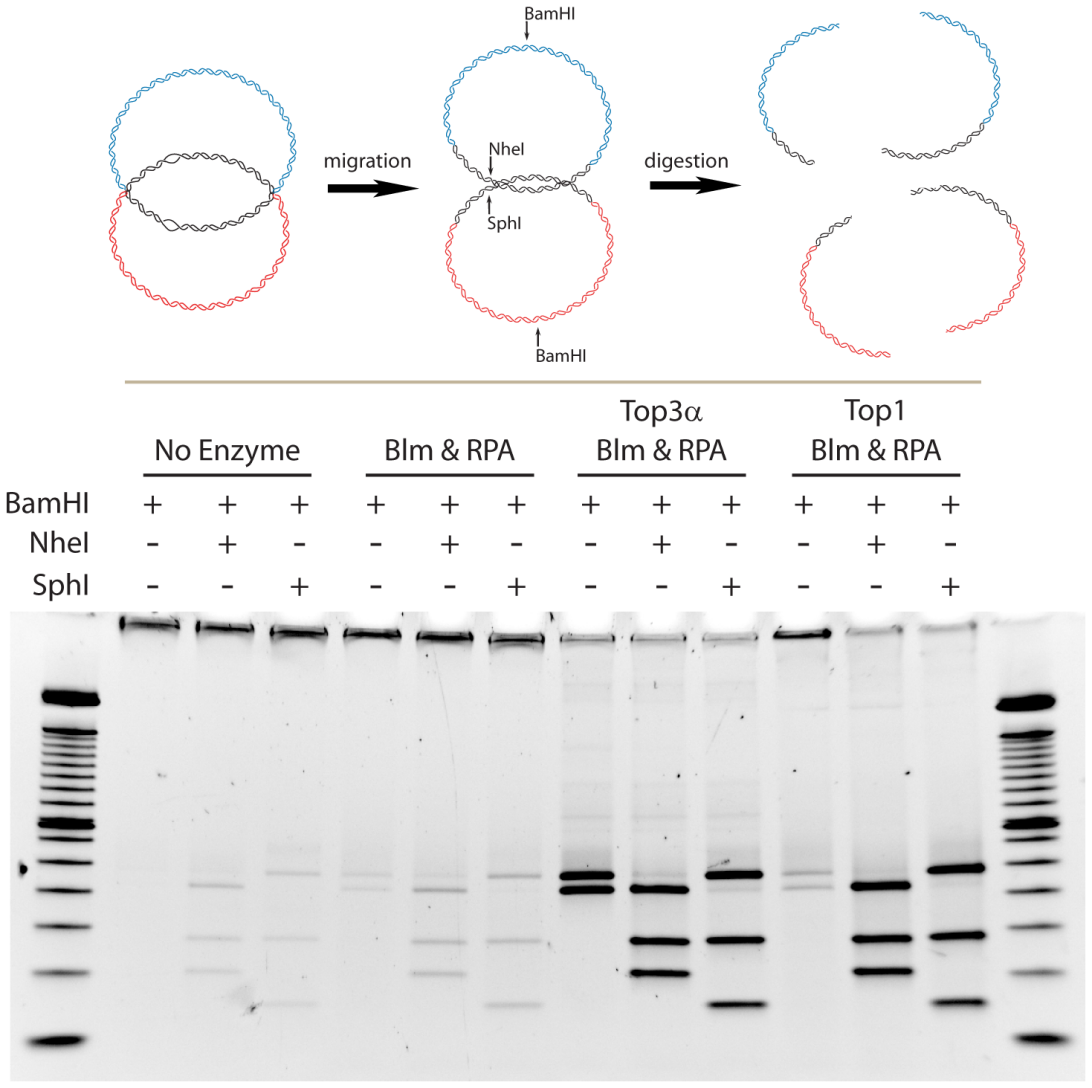
Testing for migration of the HJ using the mismatch dHJ substrate. A diagram of the reaction is at the top. Complete dHJ dissolution is indicated by the appearance of two characteristic bands after digestion with just BamHI (first lane of each set). If migration is successful, digestion with BamHI and NheI (second lane in each set) or BamHI and SphI (third lane in each set) will result in three characteristic bands. Migration can occur even if dissolution does not. Although some background is evident, it is clear that substantial migration occurs in the presence of either topoisomerase, but dissolution occurs only with Top3α.

Blm alone is not able to migrate the Holliday junction past a distance of 40 bp where the mismatch sites are located, as indicated by the lack of restriction digest products (Fig. 2). As expected, Blm in the presence of Top3α and RPA is successful at both migration and dissolution. To test if topological restriction is the reason for the lack of migration with Blm alone, we added *Drosophila* Top1, a type IB topoisomerase capable of binding to and fully relaxing duplex DNA. In the presence of Top1, Blm was able to migrate the junction past the restriction site at levels equal to Top3α and RPA, but was unable to fully dissolve the substrate. This indicates that in the presence of Top1, which is likely available in the cell, Blm can migrate a constrained HJ, but is unable to fully unlink the dHJ.

### The product of Blm & Top1 is not a hemicatenane

**Figure 3:**
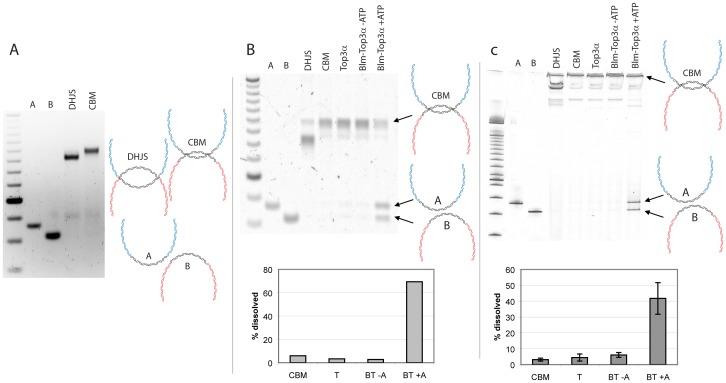
Dissolution of the CBM intermediate created by Blm and Top1. A. Creation of the CBM, a slower migrating species whose junctions are closer than the starting substrate (DHJS). B. The CBM cannot be dissolved by Top3α alone, but can be in the presence of Blm and Top3α. The results are quantified in the graph below. DNA molecules were analyzed by agarose gel electrophoresis and shown in (A) and (B). C. Same as (B) but using an acrylamide gel to enhance the resolution. The results are quantified in the graph below, and are the results of three independent trials. Error bars indicate standard deviation.

As it has been suggested that the product of Blm migration is a hemicatenane, we wanted to determine if the product of Blm and Top1 migration was a hemicatenane structure. Because the product of Blm and Top1 involves migrated junctions, we refer to this DNA structure as convergently branch migrated intermediate (CBM), in accordance with previous nomenclature [26]. If the product is a hemicatenane, Top3α alone should be able to resolve it into the two product circles. However, Top3α was unable to process the CBM into dissolution products (Fig. 3B & C). When Blm and RPA were added, recapitulating the traditional dissolvasome, then the CBM could be dissolved into the expected products (Fig. 3B & C). The reaction was also dependent on ATP, indicating that the helicase activity of Blm is important. These results indicate that some dHJ character still remains in the CBM.

**Figure 4:**
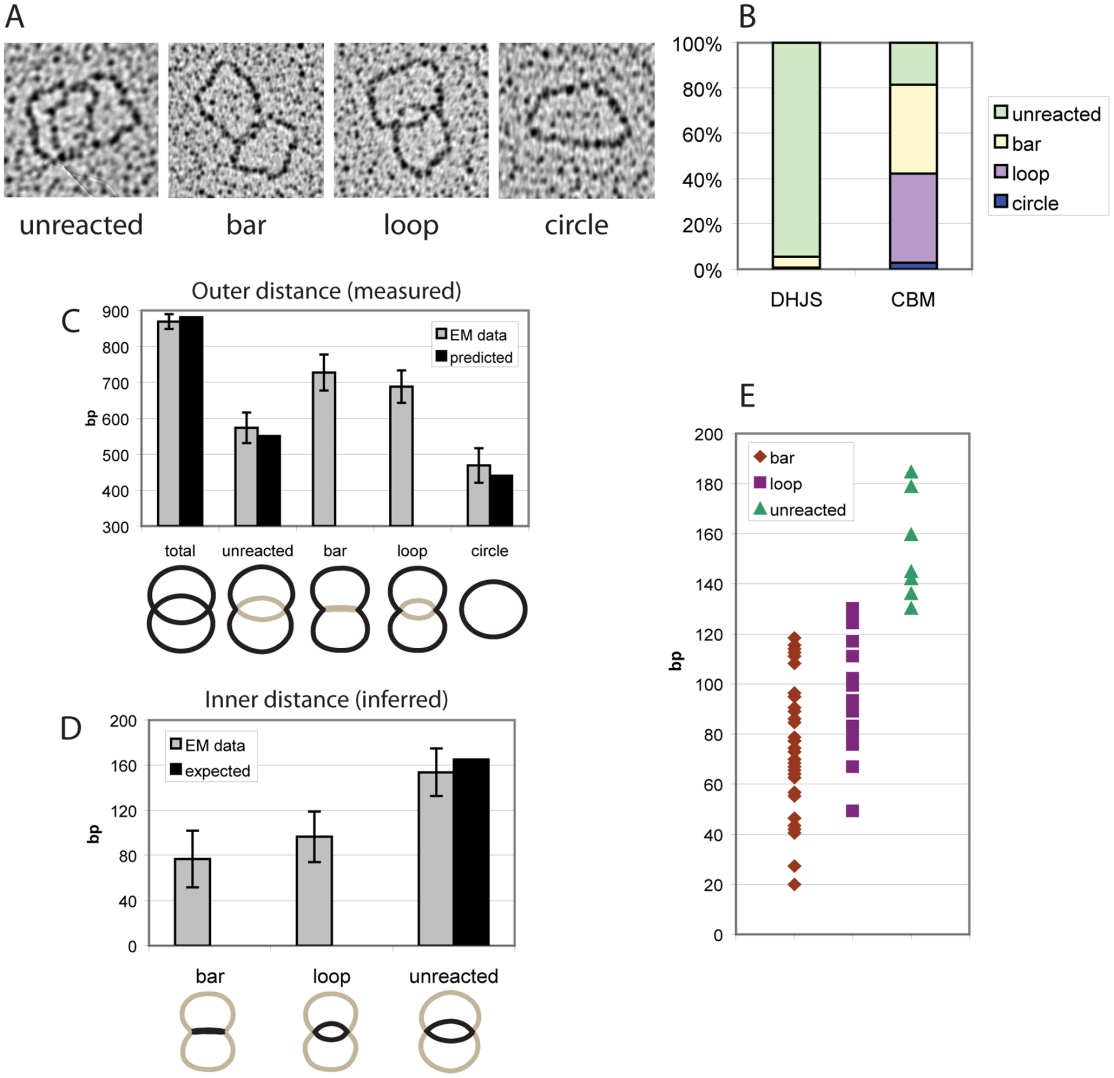
Electron microscopy of the CBM intermediate created by Blm and Top1. A. Categories of molecules observed. B. Quantification of the number of each category present before (DHJS) and after (CBM) reaction with Blm and Top1. C. Comparison of the outer-junction distances of the observed categories of molecules. “Total” is the total length of the molecule, measured from unreacted molecules. The measurements are of the lengths outside of the junctions (since the odd bar shape limits the accuracy of measurement between the junctions). Error bars indicate standard deviation. As expected, the “bar” and “loop” intermediates show an outer-junction distance between full-length (total) and unreacted. D. Comparison of the inter-junction distances of the CBM intermediates. Outer-junction distances were measured and subtracted from the total length. Error bars indicate standard deviation. Although “bar” molecules are on average closer than the “loop” molecules, they are within error of each other. E. Distribution of measured inter-junction distances shown in (D).

To determine the exact structure of the CBM, these molecules were visualized by electron microscopy. Both substrate (DHJS) and product (CBM) protein-free DNA molecules were visualized and compared. The molecules were divided into four categories based on shape: unreacted, bar, loop, and circle (Fig. 4A). The “loop” describes molecules where the junctions were closer than unreacted but retained observable space between the migratable arms, while the “bar” molecules had no separation to distinguish between the two arms and were less curved. Both the “bar” and “loop” species likely represent reaction intermediates with the dHJ undergoing limited branch migration. These species in combination account for 80% of the molecules observed in CBM (Fig. 4B). Although some “bar” structure molecules were evident in the starting material, a larger proportion of them were present after migration.

To determine the inter-junction distance of the “bar” and “loop” intermediates, the distance of the arms outside of the junctions were measured (in nm, then converted to bp) and subtracted from the length of the whole substrate. The entire length of the molecules was also measured using unreacted molecules from the starting substrate, and was found to be comparable to the predicted length. The outer-junction distances of the unreacted and circle molecules also agreed with predicted lengths (Fig. 4C).

While the bar molecules displayed slightly shorter distances, the inter-junction distances of bar and loop molecules were within error of each other (Fig. 4D). Although a variety of inter-junction distances were observed (Fig. 4E), none approached a hemicatenane, which would display an interjunction distance of zero and appear as a single junction. A well-populated minimum distance was not observed, but the average inter-junction distance was comparable to the estimated persistence length of DNA, which would equate to an inter-junction distance of 74 bp (see Discussion).

### dHJ dissolution with Blm and Top3α is highly processive

During the course of dHJ dissolution, as the junctions approach each other, a CBM-like molecule may be created as an intermediate prior to complete dissolution. If the CBM or hemicatenane state presents a kinetic barrier and is well-populated, it should appear as a slower-migrating species on a gel during the time course of dHJ dissolution.

**Figure 5:**
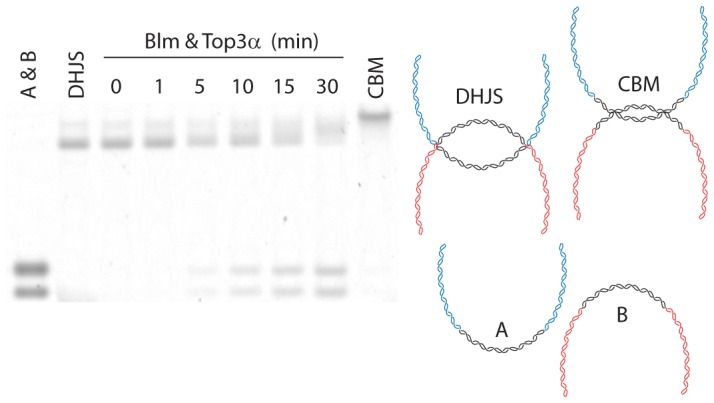
Time course of the dHJ dissolution reaction. Although products begin appearing at 5-type intermediate prior to product formation. Following reaction with the specified enzymes, the DNA was cut with BamHI and run on an agarose gel.

We analyzed by agarose gel electrophoresis the reaction products from a time course of dHJ dissolution using Blm and Top3α. We found no slower-migrating species appearing prior to the formation of dissolution products (Fig. 5), indicating that the reaction is processive and does not pause during the partially migrated or hemicatenane stage. These data demonstrate that, when the partner topoisomerase is present with Blm, both migration and dissolution occur quickly and efficiently. Therefore, Top3α is likely to be the topoisomerase together with Blm during migration as well as decatenation.

## Discussion

The process of dHJ dissolution presents a unique topological puzzle, and its disentanglement requires both the branch migration activity of Blm and the strand passage activity of Top3α. It was previously unclear whether these activities were required at the same time or could work sequentially.

It has been suggested that Top3α is not necessary for Blm's migration of the junctions, but can arrive later to simply undo the hemicatenane that forms as the result of migration [27]. Although Blm can rapidly migrate a single HJ [10], our data show that Blm cannot substantially migrate a topologically constrained junction in the absence of a topoisomerase, and that Top3α will localize to such a constrained junction, even in the absence of Blm. In addition, the Blm-Top3α complex is able to dissolve the dHJ substrate without pausing at a migration intermediate, indicating that it either involves a processive reaction that carries out migration as well as strand passage concomitantly, or one in which there is a rapid switch between these two steps.

In our study, based on visualization of molecules bound to the dHJ substrate, Top3α preferentially binds at the junction. This differs from previous results using electrophoretic mobility shift assays (EMSA). To study interactions of proteins with Holliday junctions, a static-X structure is often used, which is composed of short oligonucleotides and is non-migratable due to sequence variations of the arms. Such free-ended structures typically form the stacked, antiparallel conformation [21], [23]. However, double Holliday junctions are necessarily parallel due to their connectivity. The bending constraints of our dHJ substrate, containing a limited distance between the junctions, also cause the junctions to be more open, similar to the square planar conformation. Therefore, the junctions in our substrate have different characteristics than the oligonucleotide-based substrate used in prior EMSA assays, which are likely causing the observed differences in localization of Top3α. The most obvious difference is that the dHJ substrate is more likely to display single-strand DNA character at the junction, which is a highly preferred substrate of Top3α [15]. Double HJ's in the cell are likely to share these characteristics, which can be used to attract the repair proteins.

Blm and Top1 could partially migrate the constrained HJ, creating four classes of DNA structures. Besides the expected starting molecule (unreacted) and minimal amounts of dissolution product (circle), the two migrated species were referred to as “bar” or “loop” depending on the observed structure of their inter-junction DNA. The collapsed structure of the inter-junction DNA seen in the “bar” molecules may be due to the homology of that region of the DNA, which has been shown to produce a paranemic structure in which the two duplexes interact [28]. The increase in “bar” molecules following migration suggests that the accumulation of pressure to bend, caused by the decreasing inter-junction distance, may contribute to the formation of such a secondary structure.

The partially migrated (CBM) intermediate, created from migration of Blm and Top1, displayed a variety of inter-junction distances, ranging between 20–120 bp with an average distance of 80–90 bp, whether a “bar” or “loop” structure was formed. The relatively large range in the length distribution could be due to the resolution of the measurements by electron microscopy. In addition, it could also be due to the relatively broad length limitation for the DNA bendability. It is possible that the formation of CBM intermediates reflects constraints from DNA tension due to contorting helical segments in bringing two HJ's closer. Interestingly, the persistence length of DNA, related to the minimum length of double-strand DNA which can bend to form a complete circle, is thought to be around 50 nm, or 150 bp, in physiological salt [29]. However recent experiments with ligase-joining or single molecule FRET demonstrate that DNA circles can form readily with fragment lengths much smaller than 150 bp, up to 60 bp [30], [31]. To form a circle of this size in the area between the two junctions, the length of each arm could range between 30–80 bp, which is close to the range of average size observed in “bar” or “loop” molecules. This suggests that DNA bending may provide a limitation for the Blm/Top1 reaction and that the Blm-Top3α complex has a unique way to overcome this constraint.

Two models have been suggested for the mechanism of the Blm-Top3α dissolvasome [32]. In the “HJ Migration” model, the two enzymes are situated at the junction such that the active sites of both enzymes are able to access the DNA and both perform their functions simultaneously. However, both enzymes working at the junction would still encounter the problem of DNA bending of the inter-junction duplex. This issue is addressed in the “Unwind & Unlink” model, which proposes that Blm is first able to melt a section of DNA prior to strand passage by Top3α, thereby providing Top3α with its desired substrate and allowing the DNA to be more flexible. Both models require coordination between the enzymes to direct migration in a productive direction. Our current data do not distinguish between these two models, but do further reinforce the need for tight coordination between the two enzymes. We were unable to clearly visualize a protein complex containing both enzymes, which would provide additional clues as to the coordination of the complex. Further work is needed to determine exactly how the enzymes assemble on the DNA and able to perform this unique and elegant reaction.
